# Unravelling the evolutionary history of kisspeptin

**DOI:** 10.7554/eLife.58599

**Published:** 2020-06-15

**Authors:** Maria I Arnone, Paola Oliveri

**Affiliations:** 1 Department of Biology and Evolution of Marine Organisms, Stazione Zoologica Anton DohrnNaplesItaly; 2 Centre for Life’s Origins and Evolution, University College LondonLondonUnited Kingdom; 3 Research Department of Genetics, Evolution and Environment, University College LondonLondonUnited Kingdom

**Keywords:** neuropeptide signaling, kisspeptin, kisspeptin receptor, signal transduction, sea cucumber, physiological function, Other

## Abstract

Experiments in sea cucumbers reveal how the physiological responses regulated by a neuropeptide called kisspeptin have evolved.

**Related research article** Wang T, Cao Z, Shen Z, Yang J, Chen X, Yang Z, Xu K, Xiang X, Yu Q, Song Y, Wang W, Tian Y, Sun L, Zhang L, Guo S, Zhou N. 2020. Existence and functions of a kisspeptin neuropeptide signaling system in a non-chordate deuterostome species. *eLife* **9**:e53370. doi: 10.7554/eLife.53370

The world around us is constantly changing. As seasons shift, or as night turns to day, and food becomes more or less available, every organism must adapt their behavior and physiology to cope with its changing environment. The neuroendocrine systems play a central role in converting signals from the environment into biomolecules that can generate a response. Cells in these systems communicate by releasing various signals, including small proteins called neuropeptides. These molecules then travel towards their target cells where they bind to specific receptors and trigger a reaction that adjusts the physiology of cells and the tissues or organs they belong to (Tessmar-Raible, 2007).

A neuropeptide called kisspeptin is known to regulate fertility and reproduction in mammals (Hameed et al., 2011). The gene for kisspeptin is primarily expressed in the central nervous system, but is also active in other tissues such as the liver and heart. The gene that codes for its matching receptor shows a distinct but often overlapping pattern of expression (Bhattacharya and Babwah, 2015; Figure 1). Recent studies in adult mammals have revealed new physiological roles for this neuropeptide signaling system. For example, it has been shown that kisspeptin and its receptor regulate glucose homeostasis, feeding behavior and body mass composition by sending signals between the liver and pancreas (Wolfe and Hussain, 2018).

**Figure 1.**
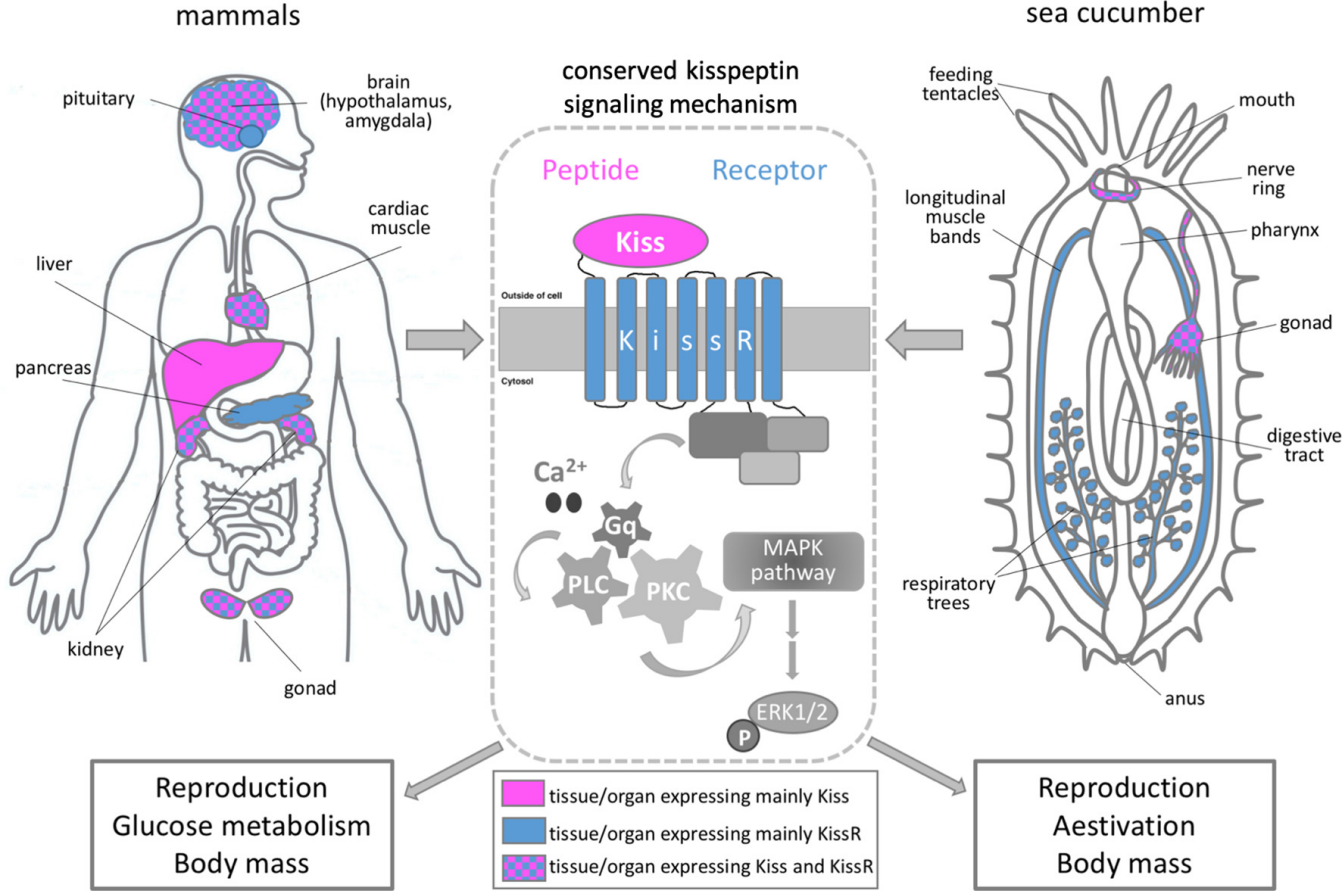
The role of kisspeptin in sea cucumbers and mammals. Schematic showing the main organs that express kisspeptin (magenta) and/or its receptor (blue) in mammals (left panel) and sea cucumbers (right panel). The binding of kisspeptin (Kiss) to its receptor (KissR) triggers an intracellular pathway which sequentially activates the signaling molecules Ca^2+^/Gq/PLC/PKC/MAPK. This stimulates a molecule called ERK1/2 which regulates a number of cellular processes, including gene expression (center panel). Wang et al. showed that the signaling cascade triggered by kisspeptin regulates similar biological processes in mammals and sea cucumbers, such as reproduction, glucose metabolism and body mass.

Image credit: Maria I Arnone.

The genes for the neuropeptide kisspeptin and its receptor have also been identified in several marine invertebrates, including members of the echinoderm family, such as sea stars, sea urchins and sea cucumbers (Elphick et al., 2018). In sea urchins, it has been reported that the gene for kisspeptin is expressed in the gut of their larvae (Wood et al., 2018). However, we know relatively little about when and where this gene is expressed in other echinoderms or about its physiological role.

Now, in eLife, Naiming Zhou from Zhejiang University and co-workers – including Tianming Wang, Zheng Cao and Zhangfei Shen as joint first authors – report experiments identifying the location and role of the kisspeptin signaling system in the sea cucumber *Apostichopus japonicus* (Wang et al., 2020). This allowed the team (who are based in China and the US) to unravel the evolutionary history of this neuropeptide signaling system, which spans over more than 540 million years.

When studying the genome of *A. japonicus,* Wang et al. identified one gene which codes for two mature neuropeptides (AjKiss1a and AjKiss1b) and two genes which encode two receptor proteins (AjKissR1 and AjKissR2). In vitro experiments in cultured human cells showed that both neuropeptides are able to bind and specifically activate either receptor. Furthermore, the receptor proteins from sea cucumbers could also be activated by kisspeptin neuropeptides from other vertebrates, including humans.

Further experiments showed that when kisspeptin binds to either of the receptor proteins in human cells, this triggers an intracellular signaling pathway that eventually activates a molecule called MAPK (Castaño et al., 2009; Figure 1). These findings were further supported by in vivo experiments in the oocytes of sea cucumbers, in which the activation of MAPK was also detected following exposure to the neuropeptide AjKiss1b. This suggests that the molecular mechanisms activated by the kisspeptin signaling system are evolutionary conserved.

Next, Wang et al. studied the expression and physiological role of kisspeptin in adult sea cucumbers. This revealed that the precursor peptide for AjKiss1 is primarily expressed in the central nervous system (nerve ring) and gonads of A. *japonicus,* and its expression levels changed significantly during the reproductive season (Figure 1). The gene for the AjKiss1R1 receptor was also active in the nerve ring and gonads, in addition to other organs, including the muscles of the body-wall and respiratory tree that are only present in sea cucumbers (Spirina and Dolmatov, 2001).

When sea cucumbers were exposed to sustained high levels of the neuropeptide AjKiss1b, this upregulated the expression of proteins involved in the metabolism of glucose and induced a dormancy-like state known as aestivation: this is characterized by extensive weight loss and degeneration of the digestive tract. This suggests that, similar to vertebrates, the kisspeptin signaling system in sea cucumbers is also involved in regulating glucose metabolism, reproduction and body mass (Figure 1). The shared role of kisspeptin in vertebrates and sea cucumbers reveals important insights into how this neuropeptide signaling system evolved.

The findings of Wang et al. demonstrate how comparing the genomes and gene roles of different organisms can unravel new aspects of animal biology that may be useful for biomedical studies. Future studies on unexplored animals will shed further light on the complex interactions that allow organisms to adapt in response to their changing environment.
